# Isolation of high-quality RNA for high throughput applications from secondary metabolite-rich *Crocus sativus* L.

**DOI:** 10.1186/s13104-022-06095-z

**Published:** 2022-06-20

**Authors:** Umer Majeed Wani, Zubair Ahmad Wani, Aabid M. Koul, Asif Amin, Basit Amin Shah, Faizah Farooq, Raies A. Qadri

**Affiliations:** Immunobiology Lab, Department of Biotechnology, University of Kashmir, Srinagar, 190 006 Jammu and Kashmir India

**Keywords:** *Crocus sativus*, RNA integrity number, High throughput sequencing, microRNA, Secondary metabolites

## Abstract

**Objective:**

Isolating high-quality RNA is a basic requirement while performing high throughput sequencing, microarray, and various other molecular investigations. However, it has been quite challenging to isolate RNA with absolute purity from plants like *Crocus sativus* that are rich in secondary metabolites, polysaccharides, and other interfering compounds which often irreversibly co-precipitate with the RNA. While many methods have been proposed for RNA extraction including CTAB, TriZol, and SDS-based methods, which invariably yield less and poor quality RNA and hence it necessitated the isolation of high-quality RNA suitable for high throughput applications.

**Results:**

In the present study we made certain adjustments to the available protocols including modifications in the extraction buffer itself and the procedure employed. Our method led to the isolation of clear and non-dispersive total RNA with an RNA Integrity Number (RIN) value greater than 7.5. The quality of the RNA was further assessed by qPCR-based amplification of mRNA and mature miRNAs such as Cs-MIR166c and Cs-MIR396a.

**Supplementary Information:**

The online version contains supplementary material available at 10.1186/s13104-022-06095-z.

## Introduction

Crocus sativus is a perennial geophyte with crimson trifid stigmas. The stigmas contain apocarotenoids such crocin, picrocrocin, and safranal, which give saffron its distinctive color, taste, and perfume [1]. It is one of the world’s most expensive spices due to its unique organoleptic properties and difficulty in cultivation, processing, and harvesting [2, 3]. Moreover, there is compelling evidence that supports the therapeutic potential of saffron [4], and due to the growing demand, it becomes important to devise a strategy to improve the quality and quantity of saffron [5].

The current scientific evidence supports the significance of microRNAs in regulating essential plant developmental processes as leaf morphogenesis, polarity [6, 7], floral differentiation, and development [8, 9]. Moreover, the microRNAs identified hitherto, have 50% targets as transcription factors out of which many miRNAs target mRNAs encoding transcription factors that regulate development [10, 11].

A stepping stone in the direction of molecular analysis involves the high-quality RNA [12] to synthesize cDNA for performing quantitative PCR, and various other experiments that rely on cDNA as a starting material. Moreover, isolating high-quality RNA becomes important while carrying out post-transcriptional studies involving microRNAs, as the preparation of a microRNA library entails meeting a specific RIN value for the RNA. To isolate RNA from different tissues of *Crocus sativus*, it is important to ensure that there is no interference of secondary metabolites, which co-precipitate with the RNA [13] and significantly reduce the quality of RNA. We isolated high-quality RNA from *Crocus sativus* samples using our modified protocol, which was developed after modifying the already published protocols originating from Chan et al. [14]. The proposed protocol is robust and ideal for isolating high-quality RNA with RIN value greater than 7.5, aimed at performing high throughput sequencing of small non-coding RNAs.

## Main text

### Methodology

After obtaining permission from the local Biodiversity Board, samples were collected from table fields of Pampore, Jammu, and Kashmir and the specimen was submitted in KASH herbarium, University of Kashmir under voucher specimen No. 4341-KASH-Herbarium. All the experiments were carried out following international guidelines.

### Reagents

Trizol Invitrogen, Extraction buffer containing (2%(w/v) SDS; 0.05 M, Tris–HCl (pH = 7.5); 0.25 M EDTA(sigma); and Polyvinylpyrrolidone (PVP) (4%); NaCl, 20 mM) was taken from the protocol proposed by Chan et al. [14] and Liu et al. [15] with slight modifications. Aside from that, phenol: Chloroform: Isoamyl alcohol (24:1v/v) (PCI 1:1 v/v), and 100% acetone were used to remove secondary metabolites that are highly soluble in acetone (Merck).

### RNA extraction

1. 50 mg of tissue was crushed to fine powder in liquid nitrogen and transferred into 1.5 ml RNAse-free tubes.
2. The samples was washed twice with 1 ml of 100% acetone, and centrifuged at 12,000×*g* for 5 min.
3. 500 μl of extraction buffer and 200 μl of β-mercaptoethanol (Sigma Aldrich) were applied to the pellet obtained. After vigorous mixing, the tubes were incubated at 25 °C for 15 min and centrifuged at 4 °C for 10 min at 12,000×*g*.
4. The supernatant (~ 300 μl) was decanted into a 1.5 ml tube and 300 µl of (1:1) Phenol: chloroform Isoamyl alcohol (24:1) was added to it and mixed thoroughly for about 2 min.
5. The tubes were centrifuged at 12,000×*g* for 10 min at 4 °C and 1/10 volumes of 3 M sodium acetate (PH 5.2) and 2.5 volumes of 100 percent ethanol were applied to the supernatant, and incubated for 1 h at 4 °C.
6. After centrifugation at 4 °C for 10 min at 12,000×*g*, the pellet was dissolved in 50 µl of Milli-Q.
7. To remove any DNA contamination, the samples were treated with DNase I (Invitrogen).
8. 300 µl of milli-Q and 200 µl (1:1) phenol: Chloroform isoamyl alcohol (24:1) was added to the tubes and mixed gently.
9. After centrifugation at 4 °C for 5 min at 12,000×*g*, the upper aqueous layer (~ 300 μl) was transferred into a 1.5 ml tubes.
10. To recover the nucleic acids, 1/10 of 3 M sodium acetate (PH 5.2) and 2.5 volumes of 100% ethanol (Merck) was added to the tubes and incubated at 4 °C for 1 h.
11. The samples were centrifuged at 4 °C for 10 min at 12,000×*g* and the pellet was dissolved in 200 μl of RNase-free water.
12. 500 μl of 10 M LiCl was added to the tubes and incubated on ice for 1 h followed by centrifugation at 4 °C for 10 min at 12,000×*g*.
13. The pellet was washed twice with 500 μl of 70% ethanol and allowed to dry in a speed vac for 2–5 min and re-suspended in 100 μl RNase-free water (Qiagen).

### Analysis of RNA

RNA isolated from different tissue of *Crocus sativus* was analyzed on 1.5% (w/v) agarose gel, and its quality were assessed by Nanodrop & Agilent TapeStation.

### cDNA synthesis and PCR

The cDNA was synthesized using RevertAid First Strand cDNA Synthesis Kit (Thermo scientific). For miRNA-specific cDNA synthesis, 2 µg of total RNA was used as a template in a reaction comprising Reverse transcriptase, reverse tubulin primer, and specific miRNA stem-loop RT primers that bind to the 3′ end of a mature miRNA, reverse transcribing the miRNA to the corresponding cDNA. For each PCR reaction, 1 μl of cDNA was added to the reaction mixture containing 5 μl of 10 × PCR buffer, 0.5 μl 10 mM dNTP Mix, 0.5 μl of 10 μM forward, 0.5 μl of Reverse Primer, and 15.3 μl of H_2_O_2_. The PCR program included: initial denaturation at 94 °C for 5 min; 35 cycles at 94 °C for 30 s, 57 °C for 30 s, and 72 °C for 40 s last extension at 72 °C for 10 min.

### Small RNA library preparation and qPCR analysis

NEBNext Multiplex Small RNA Library Prep kit New England (Biolabs) was used to prepare small RNA libraries. To evaluate the miRNA expression profile, specific cDNA was used to amplify the tubulin and candidate miRNA using primers specific to stem-loop RT primer and mature miRNA. (Additional file 1: Table S1). Tubulin was used as an internal control to normalize the expression of miRNAs. The primer efficiency was calculated using ten-fold serial dilution. For each qPCR reaction, 1 μl of cDNA diluted by a factor of 10 was mixed in a 0.2 ml RNase free tube containing 6 μl of 2X Syber green, 0.3 μl each of forward and universal reverse primer, and 4.4 ml Milli-Q water. The reaction mixture was run in lightCycler480-II (Roach) and the fold chnage was calculated using 2^−∆∆CT^ method [16].

## Results

**Fig. 1:**
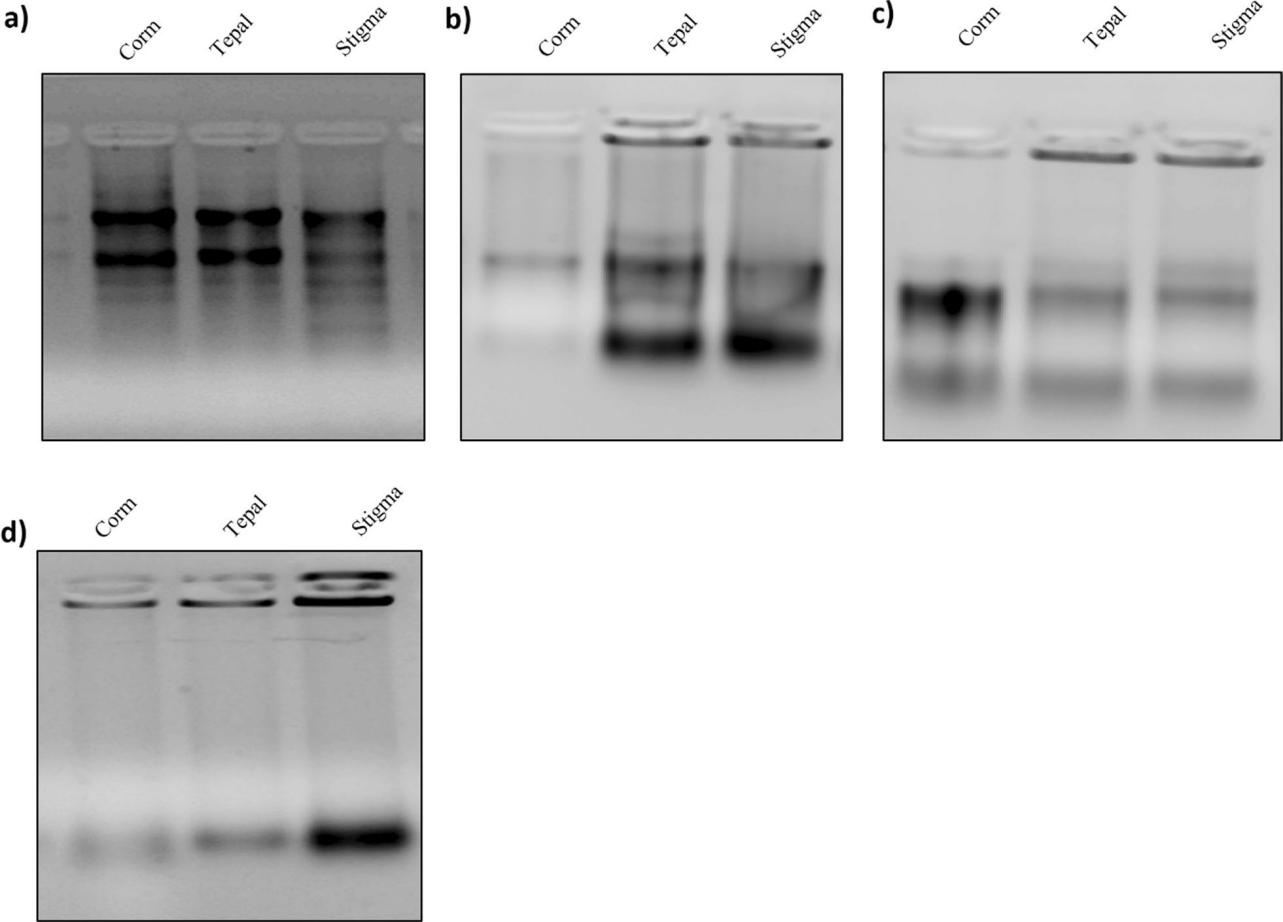
Agarose gel (1.5%) depicting RNA isolated from different tissue samples (Corm, tepal and stigma) of crocus sativus using different protocol (**a**). Trizol method (**b**). Liu et al. [15] (**c**). Chan et al. [14] (**d**). RNeasy plant mini kit. Full length gel are provided in Additional file 1: Figure S2

**Fig. 2:**
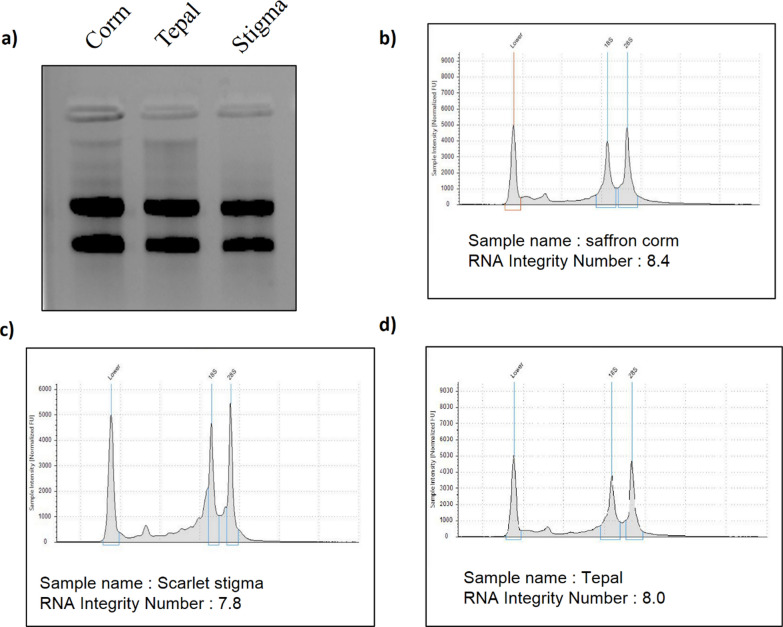
Integrity of RNA isolated using our modified protocol (**a**). 1.5% agarose gel depicting 28 s and 18 s RNA bands. Full length gels are provided in Additional file 1: Figure S3. **b**–**d** RIN value of RNA isolated from corm Tepal and Stigma respectively

While performing the extraction using the available protocols such as the Trizol method, RNeasy Plant Mini Kit, or other methods as described by Liu et al. [15] and Chan et al. [14] (Fig. 1a–d), the extracted RNA did not meet the specifications necessary for high throughput sequencing rendering the RNA unsuitable for other downstream processing. However, the modified protocol was efficient in extracting the high-quality RNA from Corm, tepal, and stigma tissues (Additional file 1: Fig. S1). The quality of RNA, based on the intensity of 28S and 18S rRNA, was clearly observed on 1.5% Agarose gel with no obvious degradation (Fig. 2a). The RIN value was found to be greater than 7.5, ranging between 7.8 and 8.4 using an Agilent Bioanalyzer (Fig. 2b–d).

Although, the RNA extracted by Trizol method was observed to be of good quality on 1.5% agarose gel (Fig. 1a). However, the RIN value of less than 6 (Additional file 1: Table S3) could not be considered good for high throughput sequencing. The spectrometric analysis of RNA extracted by modified protocol had 260/280 ratio ranging between 1.97 and 2.0 and 260/230 ratio ranging from 1.98 to 2.02 (Additional file 1: Table S2), compatible with the expected ratios for the pure RNA sample. The concentration of RNA, as obtained using, Qubit and Nanodrop, ranged from 292 to 528 ng/μl. However, when the same analysis was performed on RNA samples isolated using TRizol, Chan et al. [14] and Liu et al. [15] method, the 260/230 ratio had a marked deviation from the expected ratio, indicating the presence of secondary metabolites (Additional file 1: Tables S3–S5). Likewise, the RNA isolated by commercially available kit exhibited a deviation in 260/230 and 260/280 ratios, which was more prominent relative to other methods (Additional file 1: Table S6). It was therefore concluded that the modified protocol is significantly helpful in extracting high-quality RNA from *Crocus sativus* samples.

**Fig. 3:**
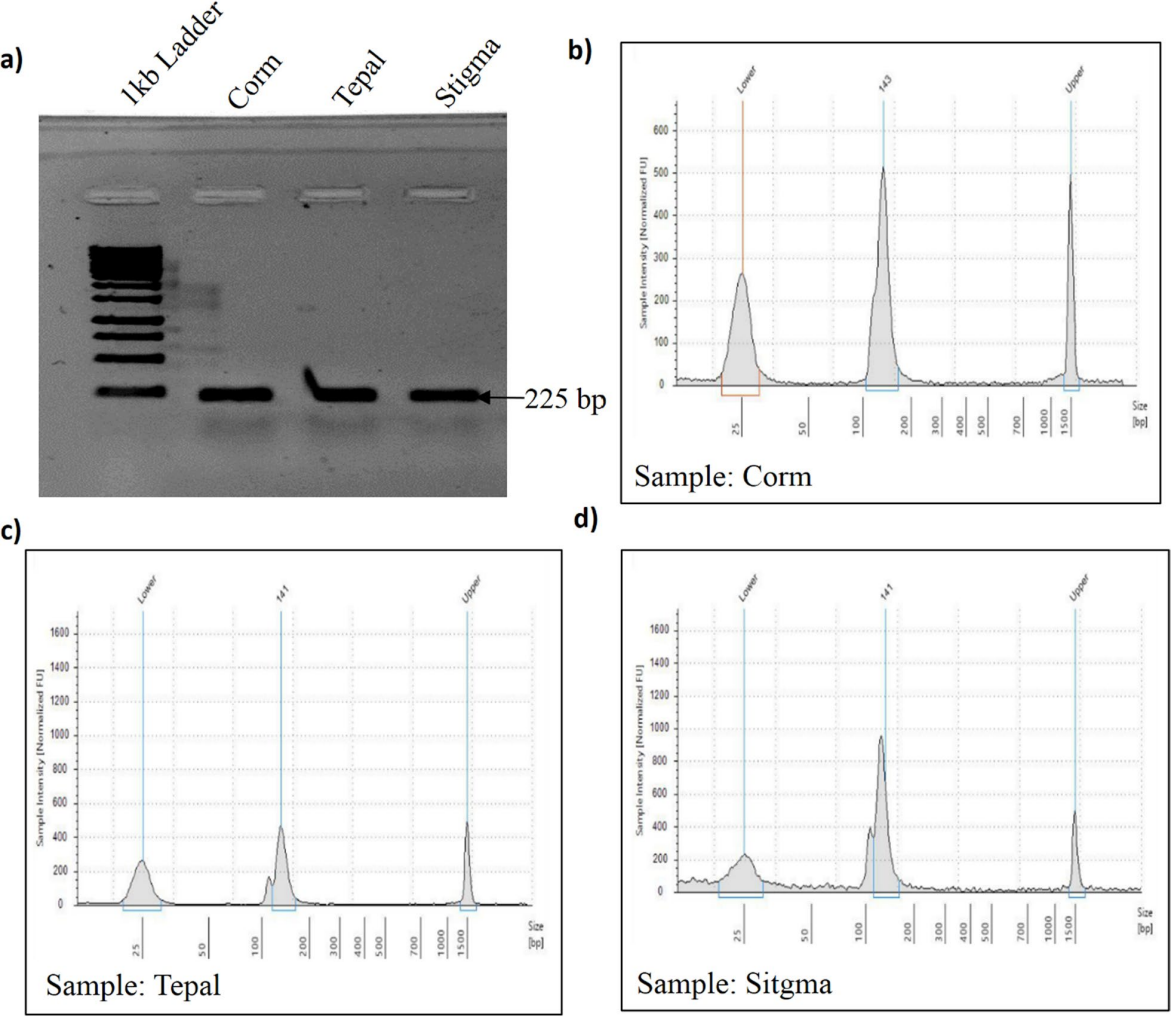
PCR analysis and Small RNA preparation from RNA isolated using modified protocol (**a**). PCR amplification of 225 bp fragment of tubulin (**b**–**d**) quality of small RNA library prepared from RNA isolated from corm, tepal and stigma. Full length gels are provided in Additional file 1: Figure S4

The quality of RNA was further validated by amplifying the tubulin gene from cDNA reverse transcribed from RNA. The band corresponding to 220 bp (Fig. 3a) indicated that the RNA was of sufficiently high quality to be explored for sRNA library preparation (Fig. 3b–d) and qPCR analysis of mature miRNAs. Moreover, the primer efficiency of tubulin, Cs-MIR166c, and Cs-MIR396a was 95–98, with an R-value of 0.99, as expected for specific primer sequences. The qPCR analysis of mature miRNAs including Cs-MIR166c and Cs- MIR396a (Additional file 1: Fig. S2a, b) depicts that the miRNA prepared from the RNA isolated by our modified protocol was of good quality.

## Discussion

For transcriptomics and other RNA-based expression studies, extracting good quality RNA with high RNA integrity value is extremely important [17]. The presence of heterogeneous secondary metabolites and polysaccharides in varied plant species poses a challenge to isolating high-quality RNA free from these interfering compounds. The problem is further compounded by unavailability of any protocol that could be universally applicable across different plant species. With the aim to isolate good quality of RNA free from interfering compounds, we developed a protocol by modifying the exsisting protocols [15, 18].

The RNA isolated using a modified protocol was better both in terms of yield and purity. The average 260/280 ratio for the RNA isolated from different tissues ranged from 1.97 to 2.0 (Additional file 1: Table S2) with maximum yield reaching as high as 528 ng/ uL indicating the suitability of this method for recalcitrant polyphenol-rich corm tissues. The same analysis when performed using other methods such as the TRizol method exhibited a significant deviation from the expected 260/280 ratio (Additional file 1: Figs. S3–S5). Further, the RIN value of isolated RNA isolated with the modified protocol was relatively higher in comparison to the lower RIN value obtained using traditional methods (Additional file 1: Table S3).

During the entire course of RNA extraction, we incorporated several modifications to minimize RNA degradation and secondary metabolite contamination. Apart from grinding the tissue in liquid nitrogen for the inactivation of RNases, we added β-mercaptoethanol, PVP, and SDS in the buffer to inhibit RNase activity, removal of phenolic compounds, and disruption of nucleoprotein complexes [19, 20]. The use of PVP proved very effective in the removal of polysaccharides, phenolic compounds, and other secondary metabolites that would otherwise get oxidized and co-precipitate with RNA. In addition, the acetone wash before lysis, significantly reduced the extracellular secondary metabolites in the lysate as acetone offered to be the solvent for dissolution of these secondary metabolites. The addition of 3 M sodium acetate and ethanol promotes the formation of ionic bonds between the negatively charged phosphate groups and positively charged ions, resulting in the neutralization, which forces the precipitation of nucleic acids out of the solution. However, this also led to the precipitation of residual DNA along with a small number of proteins. To get rid of this contamination the samples were treated with LiCl which specifically precipitates the RNA molecules. The modified protocol was both efficient and economical in isolating high-quality RNA from Crocus tissues compared to other protocols like Trizol [14, 15] and RNeasy Plant extraction Kit respectively that showed markedly blurred and irresolute bands (Fig. 2a–d). The isolated RNA can be suitably employed to carry out studies that require extremely pure and good quality RNA.

## Conclusion

The developed method is extremely ideal for high throughput sequencing of small RNAs. The method effentiently led to the isolation of clear and non-dispersive RNA with high RIN value.

## Limitations

The protocol is time-consuming in comparison to kit based protocol.

## Supplementary Information

**Additional file 1: Figure S1.** *Crocus* tissue samples (Corm, Stigma and Tepals) used for RNA isolation. **Figure S2.** Uncropped Gels of Figure 1a–d depicting cropped area (red dotted line). **Figure S3.** Uncropped Gel of Figure 2a depicting cropped area (red dotted line). **Figure S4.** Uncropped Gel of Figure 3a depicting cropped area (red dotted line).**Figure S5.** qPCR analysis of small RNA in different tissue samples of *Crocus* *sativus*. **a** Relative expression analysis of Cs-miR166c. **b** Relative expression analysis of Cs-miR396a isolated from corm, tepal and stigma respectively. **Table S1.** Primer used in semi-quantitative and real time PCR. **Table S2. **Spectrophotometric analysis of RNA isolated from different tissues of *Crocus sativus* using modified protocol. **Table S3.** Spectrophotometric analysis of RNA isolated from different tissues of *Crocus sativus* using Trizol method. **Table S4.** Spectrophotometric analysis of RNA isolated from different tissues of *Crocus sativus* using Liu et al. [14] method. **Table S5.** Spectrophotometric analysis of RNA isolated from different tissues of *Crocus sativus* using Chan et al. [13] method. **Table S6. **Spectrophotometric analysis of RNA isolated from different tissues of *Crocus sativus* using RNasy Plant kit.

## Data Availability

All data generated or analyzed during this study are included in this article and raw data is provided as a supplementary information file.

## Abbreviations

RIN value: RNA integrity number
qPCR: Quantitative polymerase chain reaction
miRNA: microRNA
PVP: Polyvinylpyrrolidone
