# Supplementary material for: Isolation of high-quality RNA for high throughput applications from secondary metabolite-rich Crocus sativus L
Source: BMC Res Notes. 2022 Jun 20;15:214. doi: 10.1186/s13104-022-06095-z (PMC9208216; doi:10.1186/s13104-022-06095-z)
Supplement: Supplementary file 1 — Additional file 1: Figure S1. Crocus tissue samples (Corm, Stigma and Tepals) used for RNA isolation. Figure S2. Uncropped Gels of Figure 1a–d depicting cropped area (red dotted line). Figure S3. Uncropped Gel of Figure 2a depicting cropped area (red dotted line). Figure S4. Uncropped Gel of Figure 3a depicting cropped area (red dotted line).Figure S5. qPCR analysis of small RNA in different tissue samples of Crocus sativus. a Relative expression analysis of Cs-miR166c. b Relative expression analysis of Cs-miR396a isolated from corm, tepal and stigma respectively. Table S1. Primer used in semi-quantitative and real time PCR. Table S2. Spectrophotometric analysis of RNA isolated from different tissues of Crocus sativus using modified protocol. Table S3. Spectrophotometric analysis of RNA isolated from different tissues of Crocus sativus using Trizol method. Table S4. Spectrophotometric analysis of RNA isolated from different tissues of Crocus sativus using Liu et al. [14] method. Table S5. Spectrophotometric analysis of RNA isolated from different tissues of Crocus sativus using Chan et al. [13] method. Table S6. Spectrophotometric analysis of RNA isolated from different tissues of Crocus sativus using RNasy Plant kit. [file 13104_2022_6095_MOESM1_ESM.docx]

**Supplementary data**

**Isolation of high quality RNA for high throughput applications from secondary metabolite rich *Crocus sativus* L.**

Umer Majeed Wani^¶^, Zubair Ahmad Wani^¶^ , Aabid M Koul, Asif Amin, Basit Amin Shah, Faizah Farooq, Raies A Qadri^*^

^Immunobiology Lab Department of Biotechnology University of Kashmir Srinagar, Jammu and Kashmir, India^

^¶ Equal contribution^

**Full length Gels**


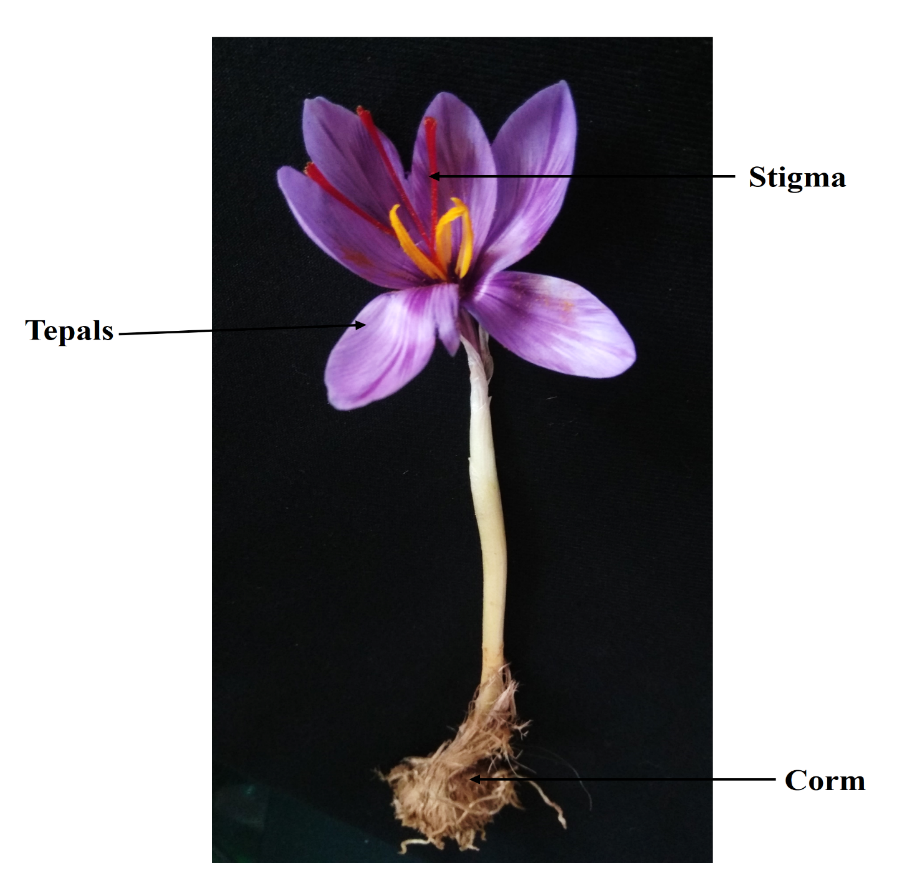


**Figure S1.** *Crocus* tissue samples (Corm, Stigma and Tepals) used for RNA isolation

^
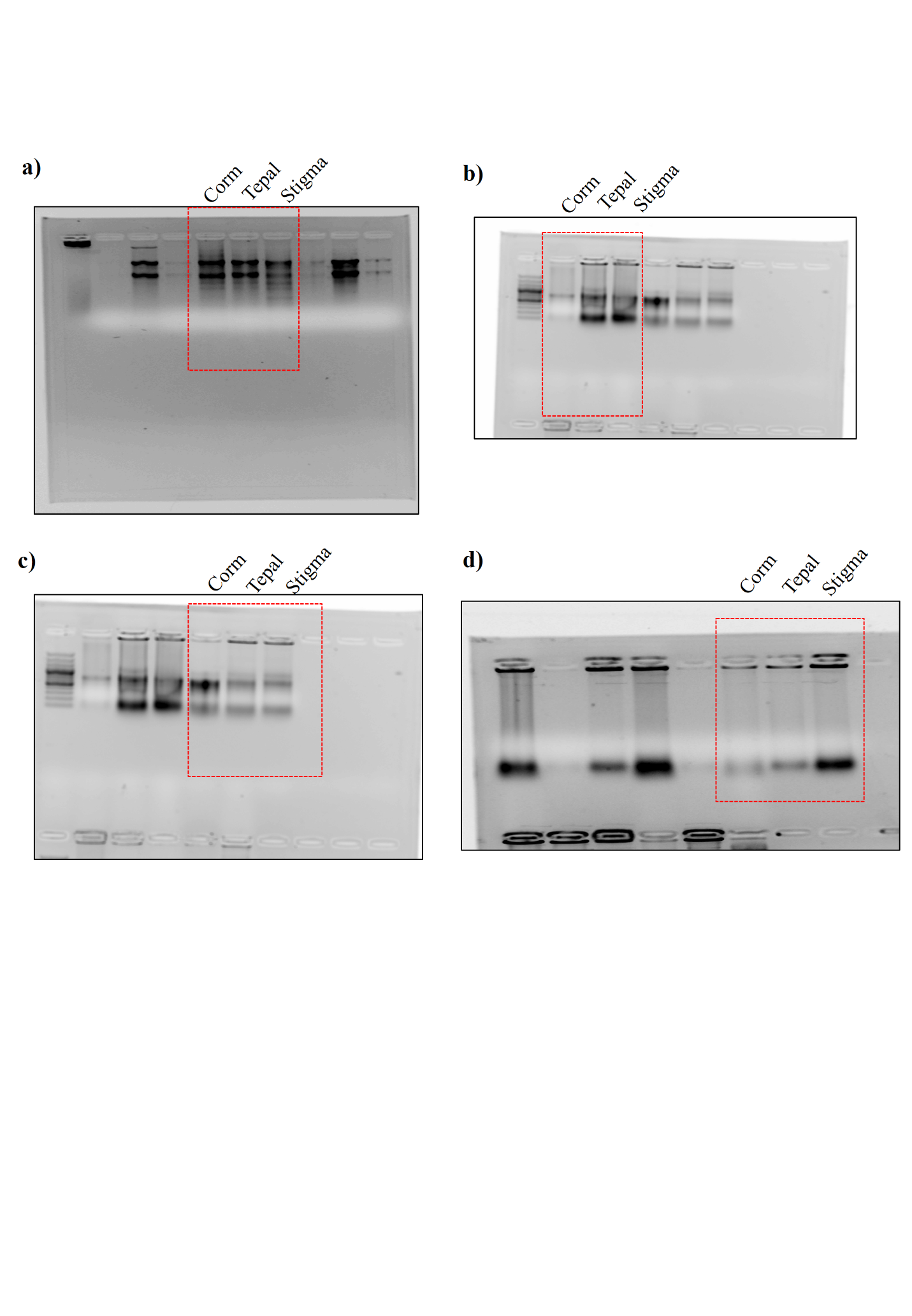
^

**Figure S2**: Uncropped Gels of Figure **1 (a-d)** depicting cropped area (red dotted line)**.**

^
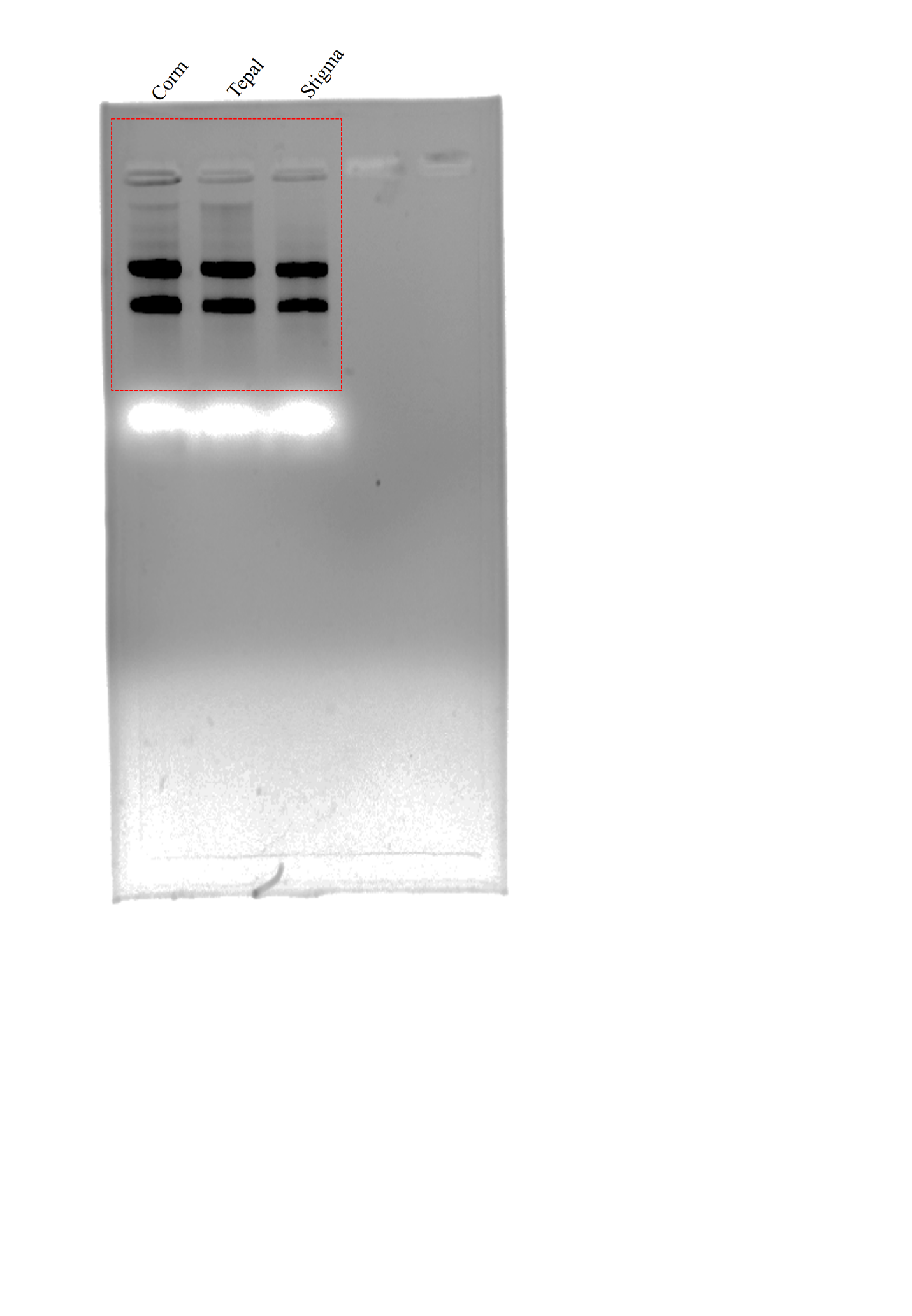
^

**Figure S3**: Uncropped Gel of **Figure 2a** depicting cropped area (red dotted line)**.**

^
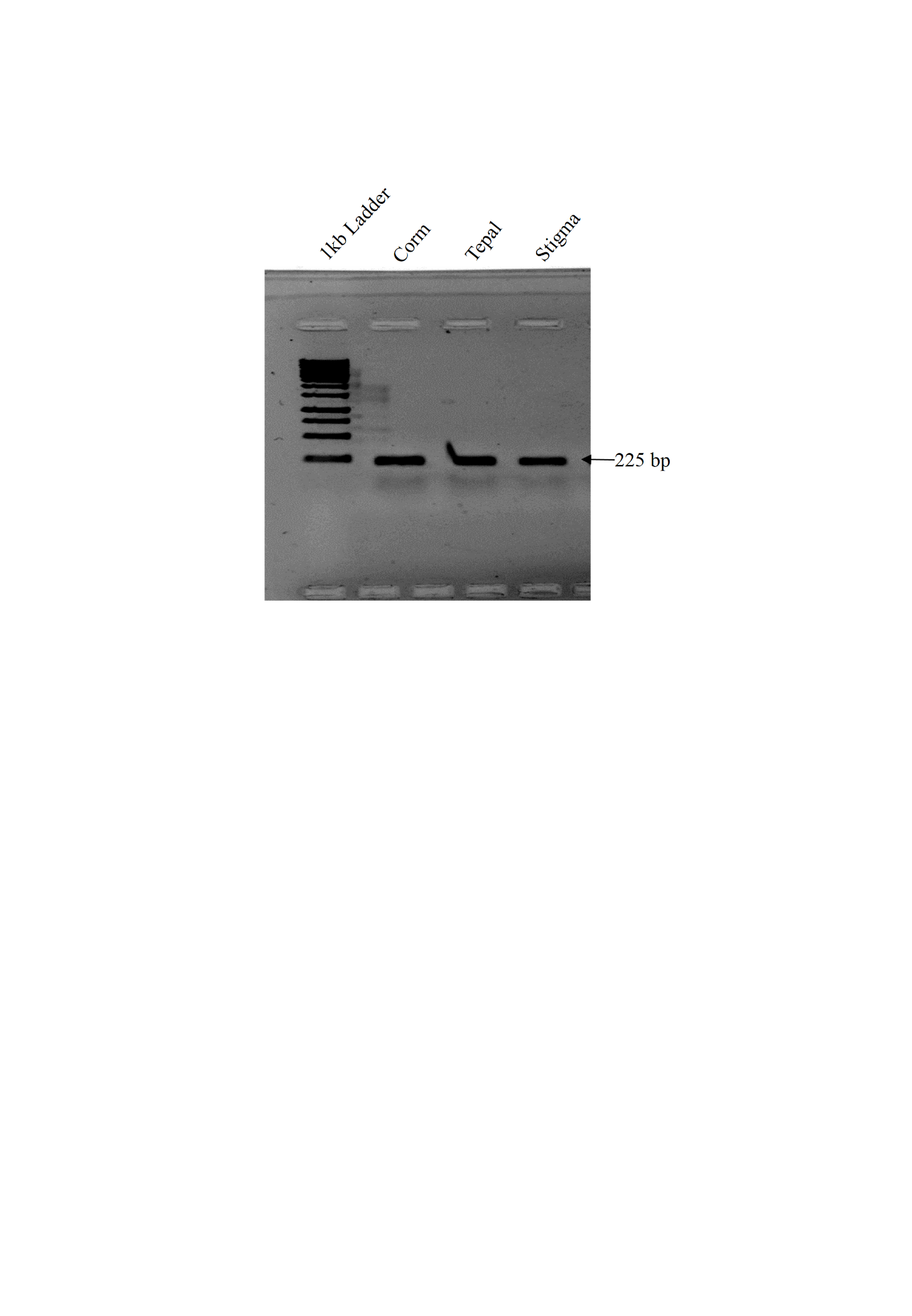
^

**Figure S4**: Uncropped Gel of **Figure 3a** depicting cropped area (red dotted line)**.**


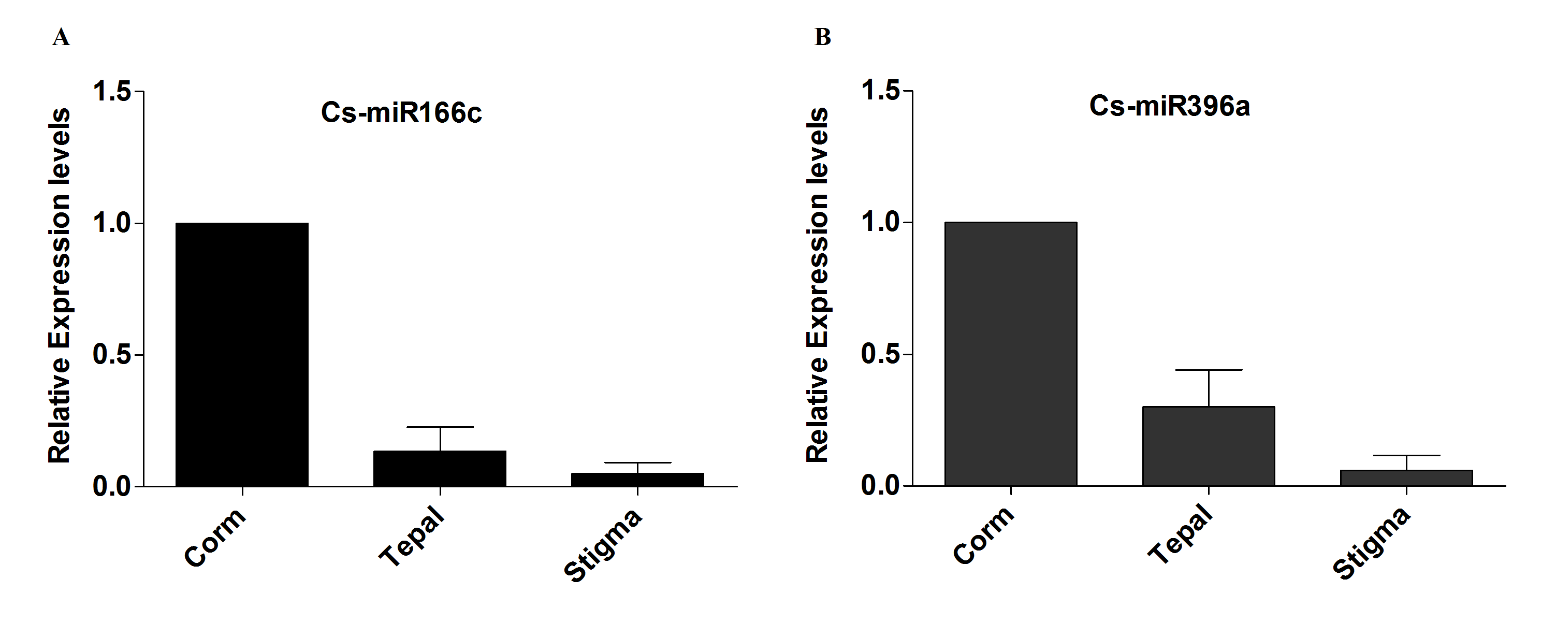


**Figure S5. qPCR analysis of small RNA in different tissue samples of *Crocus sativus* (a)** Relative expression analysis of Cs-miR166c (**b)** Relative expression analysis of Cs-miR396a isolated from corm, tepal and stigma respectively

**List of Tables**

| **Primer Name** | **Sequences 5’ to 3’** |
| --- | --- |
| **Cs-MIR****396a-F**  **Cs-MIR396a-RT** | CGGCTTCCACAGCTTTCTTGAACTG |
|  | GTCGTATCCAGTGCAGGGTCCGAGGTATTCGCACTGGATACGACCAGTTCA |
| **Cs-miR166c-F**  **Cs-miR166c-RT** | GGAATGTTGTCTGGCTCGAGG |
|  | GTCGTATCCAGTGCAGGGTCCGAGGTATTCGCACTGGATACGACCCTCGAG |
| **Universal reverse Primer** | GTGCAGGGTCCGAGGT |
| **Tubulin-F** | TTTCCAACTCGACCAGTGTC |
| **Tubulin-R** | TACTCATCACCCTCG TCACCA |

**Table S1**: Primer used in semi-quantitative and real time PCR

**Table S2:** Spectrophotometric analysis of RNA isolated from different tissues of *Crocus sativus* using modified protocol.

| **Sample Name** | **260/280** | **260/230** | **Qubit concentration (ng/µl)** | **Nano drop concentration**  **(ng/µl)** | **RIN Value** |
| --- | --- | --- | --- | --- | --- |
| Corm | 1.98±0.08 | 2.05±0.01 | 342 | 528 | 8.4 |
| Tepal | 1.97±0.05 | 2.01±0.03 | 393 | 420 | 8.0 |
| Scarlet Stigma | 1.89±0.03 | 2.0±0.04 | 401 | 441 | 7.8 |

**Table S3:** Spectrophotometric analysis of RNA isolated from different tissues of *Crocus sativus* using Trizol method.

| **Sample Name** | **260/280** | **260/230** | **Qubit concentration (ng/µl)** | **Nano drop concentration**  **(ng/µl)** | **RIN Value** |
| --- | --- | --- | --- | --- | --- |
| Corm | 1.83±0.03 | 1.70±0.09 | 292 | 230 | 6.3 |
| Tepal | 1.64±0.01 | 1.88±0.02 | 210 | 200 | 6.4 |
| Scarlet Stigma | 1.43±0.06 | 2.05±0.07 | 192 | 188 | 5.5 |

**Table S4:** Spectrophotometric analysis of RNA isolated from different tissues of *Crocus sativus* using L Liu, R Han, N Yu, W Zhang, L Xing, D Xie and DJPo Peng [14] method.

| **Sample Name** | **260/280** | **260/230** | **Qubit concentration (ng/µl)** | **Nano drop concentration**  **(ng/µl)** | **RIN Value** |
| --- | --- | --- | --- | --- | --- |
| Corm | 1.58±0.04 | 1.05±0.05 | 109 | 102 | 6.0 |
| Tepal | 1.50±0.05 | 1.21±0.03 | 142 | 153 | 5.1 |
| Scarlet Stigma | 1.47±0.01 | 0.98±0.07 | 187 | 185 | 5.8 |

| **Sample Name** | **260/280** | **260/230** | **Qubit concentration (ng/µl)** | **Nano drop concentration**  **(ng/µl)** | **RIN Value** |
| --- | --- | --- | --- | --- | --- |
| Corm | 1.49±0.10 | 1.94±0.09 | 32 | 49 | 6.3 |
| Tepal | 1.70±0.02 | 0.93±0.01 | 164 | 151 | 5.0 |
| Scarlet Stigma | 1.03±0.06 | 1.64±0.03 | 167 | 233 | 4.9 |

**Table S5:** Spectrophotometric analysis of RNA isolated from different tissues of *Crocus sativus* using K-L Chan, C-L Ho, P Namasivayam and S Napis [13] method.

**Table S6:** Spectrophotometric analysis of RNA isolated from different tissues of *Crocus*

*sativus* using RNasy Plant kit.

| **Sample Name** | **260/280** | **260/230** | **Qubit concentration**  **(ng/µl)** | **Nano drop concentration (ng/µl)** | **RIN Value** |
| --- | --- | --- | --- | --- | --- |
| Corm | 0.64±0.03 | 0.19±0.07 | 7 | 6 | 3 |
| Tepal | 0.77±0.09 | 0.13±0.01 | 6 | 0.98 | 2 |
| Scarlet Stigma | -0.10±0.07 | -0.03±0.01 | -0.97 | -1.20 | 1 |
